# Radiotherapy to the Primary Tumor: The First Step of a Tailored Therapy in Metastatic Prostate Cancer

**DOI:** 10.3390/diagnostics12081981

**Published:** 2022-08-16

**Authors:** Matteo Ferro, Felice Crocetto, Giuseppe Lucarelli, Elena Lievore, Biagio Barone

**Affiliations:** 1 Department of Urology, European Institute of Oncology (IEO) IRCCS, 20141 Milan, Italy; 2 Department of Neurosciences, Reproductive Sciences and Odontostomatology, University of Naples “Federico II”, 80131 Naples, Italy; 3 Urology, Andrology and Kidney Transplantation Unit, Department of Emergency and Organ Transplantation, University of Bari, 70124 Bari, Italy

Prostate cancer is the first most frequent cancer in men worldwide, with over 250,000 estimated new cases diagnosed in 2021 [1]. Primary treatment modalities mostly include radical prostatectomy (RP) and radiation therapy (RT). However, despite the improvements in available therapies and technologies, up to 27–53% of patients report a biochemical failure, i.e., a prostate specific antigen (PSA) level of >0.4 ng/mL or any PSA increase of >2 ng/mL higher than the PSA nadir value [2,3]. If the treatment of metastatic prostate cancer is well-established, unanswered questions remain regarding the role of the primary tumor and its potential impact on the disease. Accounting for the rationale of “hitting” the primary tumor in order to cripple the potential source of metastatic cancer cells and avoid the development of resistant clones, different randomized controlled trials (RCTs) have evaluated the role of RT in metastatic hormone-sensitive prostate cancer [4]. Indeed, while this is a common practice in other cancers (including lung, breast, renal, and ovarian cancer), in which RT was significantly associated with better overall survival (OS), currently no validated consensus is reported on local radiation therapy in metastatic prostate cancer [5,6,7].

The HORRAD trial was one of the first multicenter prospective RCT which aimed to assess the potential prolonged survival of adding local treatment to metastatic prostate cancer (mPCa). With 28 centers involved for a total of 432 patients with mPCa, assigned to RT plus androgen deprivation therapy (ADT) (intervention group) or ADT alone (control group), the study, albeit did not show a significant difference in OS and reported radiation doses not reflective of current clinical practice, unveiled a first piece of evidence on the feasibility and the potential efficacy of the treatment of the primary tumor in a metastatic setting [8].

The STAMPEDE trial was, instead, one of the largest RCT investigating the role of radiotherapy in mPCa, with 117 centers involved for a total of 2061 patients randomized to ADT versus docetaxel or RT. RT, in particular, improved failure-free survival (from 33% to 50%) but not OS in patients with high metastatic burden, defined as one or more outside vertebral bodies or pelvis metastases. Conversely, overall survival was improved in patients with a low metastatic burden (81% versus 73%), with no relevant adverse effects [9,10].

Despite the interesting results, both trials reported a few limitations which could have hampered the efficacy of the intervention. The HOORAD trial was underpowered from the standpoint of metastatic burden while the STAMPEDE trial did not evaluate the oligometastatic prostate cancer patients, in addition to the potentially limited evaluation of the metastatic burden related to not up-to-date positron emission tomography (PET) [11].

Another interesting study, currently in phase 2, is the ORIOLE clinical trial which aims to determine if stereotactic ablative radiotherapy improves oncologic outcomes in oligometastatic prostate cancer. In 54 men, progression at 6 months occurred in 19% of patients receiving RT and 61% in those undergoing observation only. The RT treatment improved, in addition, the median progression-free survival and the consolidation of prostate specific membrane antigen (PSMA) radiotracer-avid disease, decreasing the risk of new lesions at 6 months [12].

A similar study, the STOMP trial (NCT01558427), is currently ongoing, and aims to evaluate the impact of stereotactic body radiotherapy on the start of palliative ADT compared to patients undergoing active surveillance [13].

Further trials are currently ongoing as the PEACE1 trial (NCT01957436) and the SWOG/NCTN trial (NCT03678025), aiming to evaluate the role of RT on the primary tumor in a metastatic setting [14,15]. The results are however to be expected in the next decade.

The STOPCAP meta-analysis, performed by Burdett et al., systematically reviewed in 2019, prostate radiotherapy trials, reporting on PEACE-1, HORRAD, and STAMPEDE trials no overall improvement in survival while there was an overall improvement in biochemical progression and failure-free survival, with an equivalent 10% benefit at 3 years. However, the effects of prostate radiotherapy varied, based on metastatic burden. The authors concluded, therefore, that prostate RT should be considered for men with metastatic prostate cancer with a low metastatic burden [16].

Morgan et al. reported, in an interesting real-world study involving 410 patients treated with near-radical doses of EBRT (40 Gy in 15 fractions) in de novo metastatic hormone-sensitive prostate cancer as an adjunct to ADT, the efficacy and feasibility of RT in the treatment of the primary tumor in metastatic patients. In particular, at a mean of 61 months of follow up, patients receiving RT reported an overall survival at 2 and 5 years of 74.5% and 41.1%, respectively, compared to 53.1% and 25% in those with ADT only. Despite the limitations of the study, related to its retrospective and non-randomized design, this work represents the largest single-center experience of primary hormone-sensitive mPCa treated with RT, with a relatively long-term follow-up. Furthermore, the real-world experience, consistent with the data reported in the HORRAD and STAMPEDE trials, represents another strength of this study [17].

The implications of those data reported together represent an appealing perspective: patients with a low-volume metastatic disease could benefit the most from an aggressive local therapy with limited, although present, adverse effects [18]. In addition, the treatment of the primary tumor could hamper the resistance to ADT, blocking the local conversion of adrenal androgens, while, contextually, halting the local progression of the disease and, thus the development of symptoms related to primary tumor growth [19].

In this regard, the accurate identification of patients with metastatic disease, particularly with improved imaging, could further extend the potential application of primary tumor treatment in oligometastatic PCa patients, which could most benefit from a prompt and aggressive therapy [20]. Nevertheless, the limitations of novel PET/CT (positron emission tomography/computed tomography) scans, as well as magnetic resonance imaging (MRI), should also be considered. Indeed, if 18F-Sodium Flouride (18F-NaF) PET/CT report a higher specificity in the detection of bone metastasis compared to choline-based PET/CT and bone scintigraphy, its performance is still lower than whole-body MRI, with similar issues reported for lymph nodes metastases [21,22]. PSMA PET/CT scan, which utilizes as a biomarker a type II transmembrane glycoprotein normally found in the cytoplasm of prostatic cells, although reports better results in terms of metastases detection, requires larger clinical applications in order to validate its use in current urologic guidelines, in addition to increased costs [23]. Similarly, MRI suffers from a low specificity in the detection of lymph nodes metastasis, albeit machine learning protocols, as well as coupling with PET/CT, are considered among strategies to improve the detection of oligometastases [24,25].

Another interesting point of discussion is that the standard of care of STAMPEDE and HORRAD trials was ADT or ADT plus docetaxel. As reported by a recent network meta-analysis, those are an inferior treatment compared to the triplet of ADT, androgen receptor axis-targeted therapy (ARAT) and docetaxel which shows to have a 77% likelihood of being the best treatment strategy compared to ADT plus ARAT, which reached a 23% of likelihood [26]. As a result, the role of RT should be also evaluated in conjunction with ADT plus ARAT, as well as to the triplet strategy, in order to evaluate the potential improvement on overall survival.

The role of RT in mPCa is evolving and recent and ongoing trials will clarify the benefit of local therapy in this setting, in addition to significant real-world experiences. Nevertheless, further efforts are required in order to properly define the oligometastatic PCa patients and, therefore, provide a tailored therapy to those who could most benefit from the treatment of the primary tumor.
